# The Association Between Glucocorticoid Administration and the Risk of Impaired Efficacy of Axicabtagene Ciloleucel Treatment: A Systematic Review

**DOI:** 10.3389/fimmu.2021.646450

**Published:** 2021-04-20

**Authors:** Zhen Sun, RenDe Xun, MengSi Liu, XiaoQin Wu, HongTao Qu

**Affiliations:** ^1^ Hengyang Medical College, University of South China, Hengyang, China; ^2^ Department of Neurosurgery, The First Affiliated Hospital, University of South China, Hengyang, China

**Keywords:** axicabtagene ciloleucel, glucocorticoid, chimeric antigen receptor T cell therapy, neoplasms, efficacy, risk factor

## Abstract

**Background:**

Glucocorticoid is one of the common and important strategies for the treatment of chimeric antigen receptor T (CAR-T) cell therapy-related toxicity. However, there has been a theoretical concern about whether glucocorticoids use can impact the expansion of CAR-T cells and thus impair its efficacy. Hence, we reviewed studies related to the Axicabtagene ciloleucel (Axi-cel), a first-class and widely used CAR-T cell product, to elucidate the association between glucocorticoids administration and efficacy of Axi-cel.

**Method:**

We systematically searched PubMed, Embase, Web of Science, and Cochrane Library to identify studies of Axi-cel that used glucocorticoids as an intervention for the treatment of CAR-T cell-related adverse events and respectively evaluated any efficacy endpoints of intervention and controlled cohorts, published up to February 17, 2020. There were no restrictions on research type and language.

**Results:**

A total of eight studies with 706 patients were identified in the systematic review. Except for one study found that high cumulative dose, prolonged duration and early use of glucocorticoids could shorten progression-free survival and/or overall survival, and another study that found a negative effect of glucocorticoids administration on overall survival in univariate analysis but disappeared in multivariate analysis, none of other studies observed a statistically significant association between glucocorticoids administration and progression-free survival, overall survival, complete response, and overall response rate.

**Conclusion:**

Our study indicated that the association between glucocorticoids therapy and the efficacy of CAR-T cell may be affected by cumulative dose, duration, and timing. There is currently no robust evidence that glucocorticoids can damage the efficacy of CAR-T cell, but the early use of glucocorticoids should be cautiously recommended.

## Introduction

CAR-T cell therapy involves the modification of human autologous or allogeneic T cells to target specific antigens so as to delay or cure diseases (1, 2). In recent years, this therapy has made rapid progress in the treatment of lymphoma and leukemia, and has become the most promising treatment for patients with relapsed or refractory hematologic malignancies. However, compared with traditional chemotherapy and autologous stem cell transplant, it has some unique side effects, of which cytokine release syndrome (CRS) and immune effector cell-associated neurotoxicity syndrome (ICANS) are the most common and concerning adverse effects (3). Many patients have to be admitted to the intensive care unit because of these two side effects, which can greatly prolong hospitalization and increase costs, and sometimes even fatal.

The pathophysiology of CRS has been extensively studied, which is thought to be caused by the release of inflammatory cytokines due to the activation of CAR-T cells and other immune cells such as monocytes or macrophages, and tocilizumab, an interleukin-6 receptor inhibitor, has been approved as a therapeutic strategy (4–8). However, the exact mechanism of ICANS remains largely unclear, the endothelial activation and increased permeability of the blood–brain barrier, as well as elevated inflammatory factors such as interleukin (IL)-1, IL-6, IL-10, C-reactive protein, ferritin and interferon-γ are thought to play a key role (9–14). Currently, several toxicity management guidelines for CAR-T cell therapy recommend the use of glucocorticoids for CRS that is refractory to anti-IL-6 therapy and grade 1–4 ICANS, although this has not been formally approved (15–18). However, some studies suggested that the glucocorticoids may blunt the expansion and persistence of CAR-T cells *in vivo*, and impair therefore anti-tumor activity (11, 19, 20).

Axicabtagene ciloleucel (Axi-cel) is a second-generation CAR-T cell that uses CD28 as co-stimulation and transmembrane domain and targets CD19, which was commercially approved by the US Food and Drug Administration in 2017 for the treatment of r/r aggressive non-Hodgkin’s lymphomas (21). In patients treated with Axi-cel, ICANS tends to be severe, with approximate 30% of patients experiencing grade 3 or higher ICANS, which means that there is a high probability of using glucocorticoids in patients receiving Axi-cel (22–24).

At present, whether glucocorticoids have negative effects on the efficacy of Axi-cel remains to be determined, which puts clinical decision makers in a dilemma when facing patients with ICANS. Therefore, with the increasing popularity of Axi-cel as a post-market product, it is urgent to conduct a study to clear the performance of glucocorticoids in the treatment of Axi-cel. Our study aimed to systematically review all published literature on glucocorticoids administration for Axi-cel-related to adverse effects and analyze the effects of glucocorticoid on efficacy of Axi-cel.

## Method and Materials

The systematic review followed the Preferred Reporting Items for Systematic Reviews and Meta-analyses Statement, and the protocol was enrolled in the International Prospective Register of Systematic Reviews (CRD42020213716).

### Search Strategies

We used “axicabtagene ciloleucel”, “Axi-cel”, “kte c19”, “kte c19 car”, “ktec19”, and “yescarta” as search terms to search for all Axi-cel related literature in PubMed, Embase, Cochrane library and Web of Science. Databases were searched on October 7, 2020 and updated on February 17, 2021. We also reviewed the reference lists of related reviews and included articles. There was no language limit.

### Eligibility Criteria

Studies that met the following criteria were considered for inclusion: (1) All types of clinical studies, including controlled trial, single-arm trial, retrospective study and prospective study; (2) Patients treated with Axi-cel for relapsed or refractory large B-cell lymphoma (LBCL); (3) Some patients were treated with glucocorticoids for adverse effects following Axi-cel injection; (4) A portion of patients did not use glucocorticoids after receiving Axi-cel; (5) Any one of efficacy was respectively evaluated in the glucocorticoid group and the non-glucocorticoid group, including overall response rate (ORR), complete response rate (CRR), progression-free survival (PFS), and overall survival (OS), or reported the qualitative or quantitative impact of glucocorticoid on efficacy of Axi-cel.

Studies that fell into any of the following categories were excluded: (1) Studies without original data, including reviews, comments, editorials, and meta-analysis; (2) Incomplete data or unpublished studies, including conference abstracts, study protocols, gray literature, and study data not available; (3) Animal, cell trials and other studies not performed on humans; (4) Repeated publication or studies that reused published data; (5) Studies in which fewer than two samples in intervention cohort or controlled cohort.

### Data Extraction

Two reviewers independently screened the literature and performed data extraction, and then cross-checked included studies and extracted data. The reason for exclusion was recorded and any discrepancy that arose between the two reviewers was resolved through discussion with the other authors. Data extracted included the first author, publication year, the number of patients, age, gender, pathological type, Axi-cel dose, lymphodepletion regimen, number of patients with CRS and ICANS, number of patients receiving glucocorticoids after Axi-cel infusion, dose, duration and timing of glucocorticoids, guidance on managing CRS and ICANS, and the respective responses (ORR, CRR, stable disease and progressive disease) of patients with and without glucocorticoid. The Newcastle-Ottawa scale (NOS) was used to assess the methodological quality of eligible studies, including selection, comparability and outcome.

### Outcome Measures

The primary endpoints were the PFS and OS. The secondary endpoints were the ORR (the percentage of patients with partial and complete response in all patients) and CRR (the percentage of patients with complete response in all patients). The ORR and CRR were calculated based on the best response achieved after treatment with Axi-cel.

## Results

### Study Characteristics

**Figure 1:**
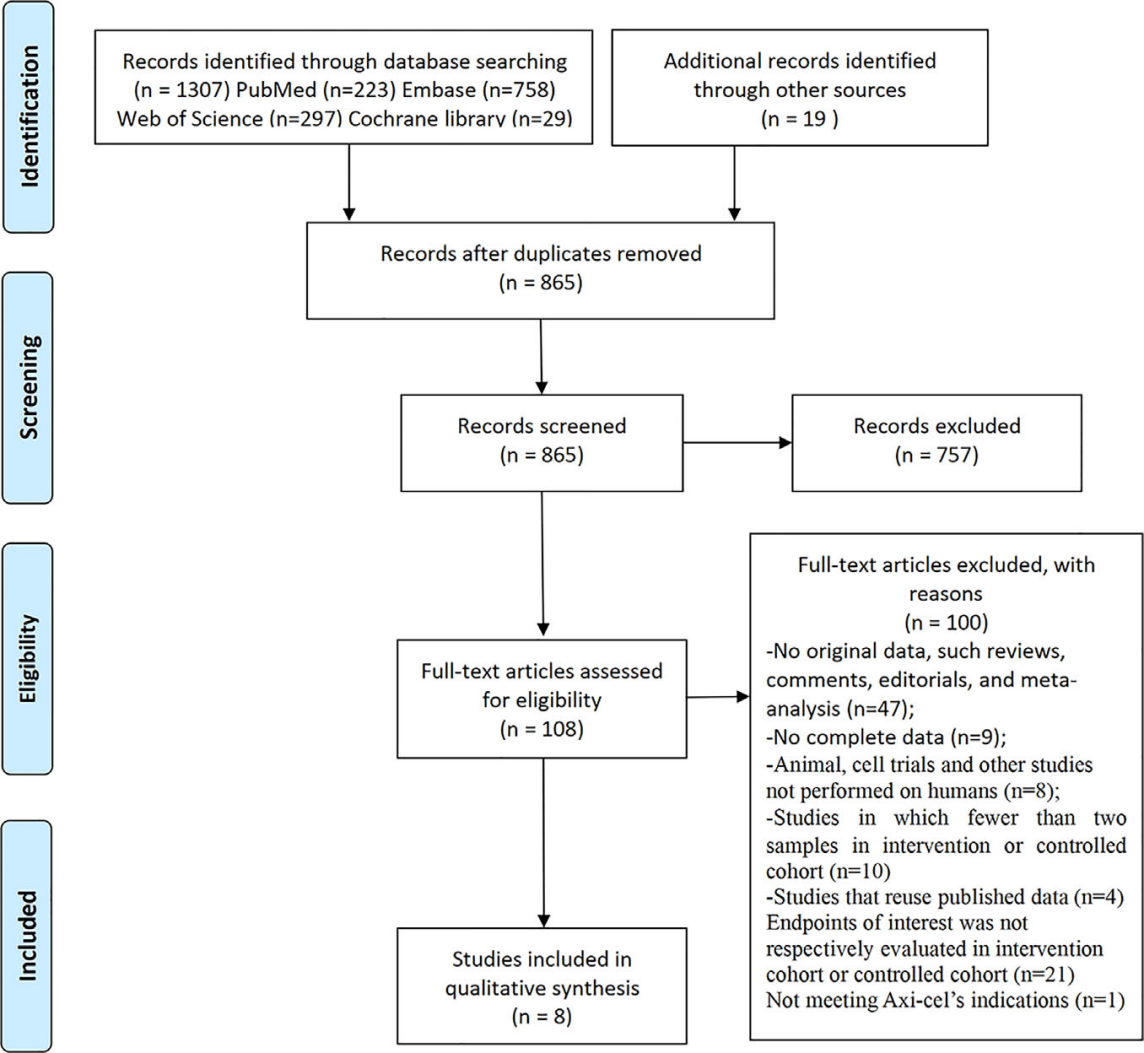
Flow diagram of the study select process.

A total of 1,307 papers related to Axi-cel were identified following our initial literature search. After removing duplicated literature and screening title/abstract and full text by two reviewers, a total of eight studies were included in the systematic review (**Figure 1**).

There was only one eligible controlled study that specifically investigated patients receiving glucocorticoids as an intervention after Axi-cel therapy, which may be due to the limited clinical experience and treatment options available for ICANS management and the need for emergency treatment following adverse reactions in patients (25). Therefore, all the studies we included were retrospective studies or case reports.

The population included 398 (56.4%) patients with diffuse large B cell lymphoma (DLBCL), 140 (19.8%) patients with transformed follicular lymphoma (tFL), 42 (5.9%) patients with primary mediastinal B-cell lymphoma (PMBCL), 19 (2.7%) patients with high-grade B cell lymphoma (HGBCL), five (0.7%) patients with transformed marginal zone lymphoma (TMZL), two (0.3%) patients with Richter’s syndrome (RS), and 100 (14.2%) patients only reported as LBCL. The median age of patients in four studies was less than 60 years, and in the remaining four studies it was greater than or equal to 60 years. All studies that reported the lymphodepletion regimen used the fludarabine 30 mg/m^2^/d × 3 days and cyclophosphamide 500 mg/m^2^/d × 3 days as in ZUMA-1 trail. Patients received Axi-cel injection at a target dose of 1–2 × 10^6^ CAR T cells/kg in all studies that reported the dosage. Only two studies reported the cumulative dose and duration of glucocorticoids, and three studies reported timing of glucocorticoids use. The toxicity management strategies reported in different studies were different, including Axi-cel’s risk evaluation and mitigation strategy, Lee scale, and independent institutional guidelines. More detailed information about the included studies is shown in **Table 1**.

**Table 1:** The baseline characteristics of the included studies.

First author (year)	No.	Median age (range)-yr.	Male	Histological type (s)	CAR-T cell dose	Chem-o	No. of using steroid	Dose and duration of steroid	Timing of steroid	Toxicity treatment guide	Efficacy response	ICANS (any grade)	CRS (any grade)
Holtzman et al. (26)	45	60 (26–75)	22	35: DLBCL 7: tFL 3: PMBCL	NP	NP	23	a	NP	REMS	22: CR 16: PR/SD 5: PD	25	NP
Nastoupil et al. (24)	298	60 (21–83)	192	203: DLBCL 76: tFL 19: PMBCL	NP	Flu/Cy	149	NP	NP	NP	175: CR 50: PR	189	251
Sesques et al. (27)	28	59 (27–72)	16	17: DLBCL 8: tFL 3: PMBCL	NP	Flu/Cy	13	NP	Median time of day 8 (6–13)	NP	13: CR 5: PR 10: SD/PD	9	26
Neelapu et al. (22)	101	58 (23–76)	68	77: DLBCL 16: tFL 8: PMBCL	2× 10^6^ CAR T cells/kg	Flu/Cy	27	NP	NP	CRS: Lee scale ICANS: institutional guideline	55: CR 28: PR 11: SD 5: PD	65	94
Locke et al. (28)	7	46 (29–69)	5	DLBCL	1–2× 10^6^ CAR T cells/kg	Flu/Cy	4	NP	NP	NP	4: CR 1: PR 1: SD	7	6
Jacobson et al. (23)	122	62 (21–79)	192	57: DLBCL 33: tFL 8: PMBCL 17: HGBCL 5: TMZL 2: RS	NP	NP	65	14% of patients receiving a high dose^*^	NP	NP	61: CR 24: PR 3: SD	85	144
Ghafouri et al. (29)	5	59 (28–76)	NP	2: DLBCL 2: HGBCL 1: PMBCL	NP	NP	2	NP	Days 4 and 7	NP	3: CR 1: SD 1: PD	2	2
Strati et al. (25)	100	60 (18–85)	74	LBCL with 77 DLBCL/HGBCL	NP	NP	60	b	I	institutional guidelines	55: CR	NP	NP

LBCL, large B-cell lymphoma; DLBCL, diffuse large B cell lymphoma; tFL, transformed follicular; PMBCL, primary mediastinal B-cell lymphoma; HGBCL, high-grade B cell lymphoma; TMZL, transformed marginal zone lymphoma; MM, multiple myeloma; RS, Richter syndrome; CAR-T, chimeric antigen receptor T; NP, not provided; CR, complete response; PR, partial response; PD, progressive disease; SD, stable disease; CRS, cytokine release syndrome; ICANS, immune effector cell-associated neurotoxicity syndrome; Flu/Cy, fludarabine at 30 mg/m^2^ and cyclophosphamide at 500 mg/m^2^ on days -5, -4, and -3; CARTOX, CAR-T-cell-therapy-associated TOXicity; REMS, Axi-cel’s risk evaluation and mitigation strategy. Institutional guidelines mean the toxicity management guidelines developed by the institution and described in the original text.

a: equivalent to 221 mg of dexamethasone (range, 52–1,630) for a median duration of 12.5 days (range, 4–27).

b: equivalent to 186 mg of dexamethasone (range, 8–1,803) for a median duration of 9 days (range, 1–30).

Ⅰ: Forty-five within the first 7 days and 15 beyond day 7.

*more than 40 mg dexamethasone or equivalent per day.

All studies were independently evaluated for study quality by using NOS (cohort studies). Because in all studies, whether patients used glucocorticoids depended on CAR-T cell-related adverse events, the selection of the nonexposed cohort of NOS was rated as 0. Overall study quality was rated as moderate quality, which is depicted in **Table 2**.

**Table 2:** The quality assessment of included studies.

Study	Selection				Comparability	Outcome			Quality score
	Representativeness of the exposed cohort6	Selection of the nonexposed cohort	Ascertainment of exposure	Demonstration that outcome of interest was not present at start of study	Comparability of cohorts on the basis of the design or analysis	Assessment of outcome	Was follow-up long enough for outcome to occur	Adequacy of follow up of cohorts	
Holtzman	★		★	★	★	★	★	★	7
Nastoupil	★		★	★	★	★	★	★	7
Sesques	★		★	★	★	★		★	6
Neelapu	★		★	★	★	★	★	★	6
Locke			★	★		★	★	★	5
Jacobson	★		★	★	★	★	★	★	7
Ghafouri	★		★	★	★	★		★	6
Strati	★		★	★	★	★	★	★	7

### Progression-Free Survival

A total of four studies investigated the effects of glucocorticoids use on PFS (24–27). Both Sesques et al. and Nastoupil et al. found no association between glucocorticoids use and PFS (24, 27). Holtzman et al. also assessed the effects of duration (P = 0.32), total dose (P = 0.59), average daily dose and initial high-dose pulsing of glucocorticoids on PFS, however, no specific cut-off value was provided (26). Strati et al. found no association between PFS and glucocorticoids use (P = 0.13), but when the cumulative dose and duration of the glucocorticoids were considered (cut-off values: 186 mg, 9 days, respectively), higher dose led to shorter PFS (p = 0.005), prolonged duration did not affect PFS (p = 0.12), and patients who early used glucocorticoids within 7 days tended to have worse PFS (p = 0.07) (25).

### Overall Survival

As with PFS, the same four studies evaluated the impact of glucocorticoids on OS (24–27). In study by Sesques et al., the HR of the glucocorticoid group compared with the non-glucocorticoid group was 1.09 (95%CI: 0.43–2.70, P = 0.85) (27). Nastoupil et al. found a statistically significant negative effect on OS in univariable analysis (P = 0.04), while disappeared in multivariate analysis (HR, 1.3; 95% CI, 0.8 to 2.2; P = 0.2) (24). Results of Holtzman et al. still suggested that glucocorticoids use did not affect OS, regardless of duration (P = 0.32), total dose (P = 0.58), average daily dose and initial high-dose pulsing (26). The study by Strati et al. showed conflicting results. They found that glucocorticoids use could significantly reduce the OS (p = 0.006). In addition, prolonged use (p = 0.003), higher dose (p <0.001), and earlier use (p = 0.005) were all associated with poorer OS (25).

### Complete Response Rate

Nastoupil et al. reported that glucocorticoids did not affect CRR of patients, but no further information was provided (24). Holtzman et al. and Strati et al. respectively used a median total glucocorticoid dose equivalent to 221 mg of dexamethasone (from 52 to 1,630) for a median duration of 12.5 days (from 4 to 27) and 186 mg of dexamethasone (from 8 to 1,803) for 9 days (from 1 to 30), both of whom assessed the effects of cumulative dose and duration of glucocorticoids on the CRR, Holtzman et al. also investigated the initial high-dose pulsing and average daily dose, and Strati et al. also studied the timing of glucocorticoids, none of which showed a statistical association between glucocorticoids and CRR (25, 26). In the phase-1 ZUMA-1 trial, three patients achieved complete response at 12 months, of whom one patient received glucocorticoids for the management of both CRS and ICANS (28).

### Overall Response Rate

There were four studies that reported the ORR in the non-glucocorticoid group and glucocorticoid group respectively, and no significant differences were found (22, 23, 28, 29). The two largest studies conducted by Neelapu et al. (83.8% vs 77.8% in non-glucocorticoid group and glucocorticoid group, respectively) and Jacobson et al. (70.2% vs 69.2% in non-glucocorticoid group and glucocorticoid group, respectively) did not show evidence that glucocorticoids affect the ORR. However, of note, the glucocorticoid groups generally tended to have a lower ORR. In the phase-2 ZUMA-1 trial, the ongoing ORR at 12 months with the glucocorticoid group and the non-glucocorticoid group were 33% (95%CI: 17–53%) and 45% (95%CI: 34–57%), respectively, which was not statistically different (22). Study with median start day 8 (from 6 to 13) by Sesques et al. found that ORs for ORR at months 1 and 3 were 0.61 (0.20–1.84) and 1.63 (0.53–4.96), respectively, which indicated that the duration of ORR in 3 months in the non-glucocorticoid group tended to be shorter than that in the glucocorticoid group, but the analysis of this study included 54.1% patients with Tisa-cel (27).

## Discussion

This is the first systematic review to specifically investigate the impact of glucocorticoids use on the efficacy of CAR-T cell therapy. Because of the structural and immunological differences between various CAR-T cell types, we only focused on the widely used CAR-T cell product Axi-cel as the study object.

Our study found that if only according to the glucocorticoids use for investigating its impact on the efficacy of CAR-T cell therapy generally drew negative conclusions. When the cumulative dose, duration and timing of glucocorticoids were further analyzed, a study found that high-dose glucocorticoids were associated with shorter PFS, and the high dose, prolonged duration, and timing all significantly impacted OS of patients (25). But another study of which major drawback was the undefined cut-off value indicated that the dose and duration of glucocorticoids had no effect on PFS and OS (26). We compared guidelines on managing ICANS and CRS of the two studies, and found that the study showing positive results tended to use glucocorticoids in the presence of mild ICANS and CRS, while the latter study had a higher threshold for glucocorticoids use, only moderate toxicities or worse were considered, which may have contributed to the conflicting results of the two studies, namely, it was important to judge the timing of glucocorticoids use based on the toxicity grade. In the ZUMA-1 cohort 4 reported by Topp et al., patients received early glucocorticoids administration starting at grade 1 ICANS and grade 1 CRS if no improvement was achieved after 3 days of supportive care. Compared with cohorts 1 and 2 of ZUMA-1, a larger proportion of patients received glucocorticoids therapy (73% vs 27%). Their results showed that the PFS, CRR, ongoing response and CAR T cell expansion were similar to cohorts 1 and 2. More importantly, the proportions of patients with severe CRS and ICANS were significantly reduced. However, the study did not compare the patients receiving glucocorticoids with those not receiving glucocorticoids within the cohort, and did not evaluate the comparability of the dose and duration of glucocorticoids between different cohorts (30).

Current limited evidence showed that glucocorticoids had no effect on CRR or ORR, regardless of the duration, total dose, and timing of administration. Although there is an established theoretical concern that glucocorticoids could impair the profile of CAR-T cells, it is possible that this effect can inhibit the excessive activation of immune cells without damaging the anti-tumor activity of CAR-T cells *in vivo* if the dose and duration are appropriate (31, 32). Another possible reason for this result was that CAR-T cells persistence was sufficient for most patients to reach ORR or CRR, despite the glucocorticoids could blunt expansion and duration of CAR-T cells (20). Hence, considering that patients with ORR have a high recurrence rate in a short time, it is necessary to combine multiple efficacy endpoints to draw accurate conclusions.

To date, the management for ICANS is nonspecific, and primarily represented by glucocorticoids, supportive care, and antiepileptics. Different studies had conflicting results on the association between ICANS and efficacy of Axi-cel therapy, therefore, although most ICANS events were reversible, the indirect effects of ICANS on Axi-cel therapy could not be ignored, and proper prophylaxis treatment may be necessary for patients at high risk of ICANS (26, 33). Several studies attributed the decreased severe CRS and ICANS rate to earlier and more systematic intervention of tocilizumab and corticosteroids, which provided evidence to support the use of glucocorticoids, however, none of these studies specifically analyzed the effects of glucocorticoids timing on CAR-T cell efficacy (22, 24, 27). Considering the findings of Strati et al., early use of glucocorticoids or as a preventive management formulation requires more careful consideration (25). More studies were needed to determine the optimal timing of glucocorticoids use, so as to find a balance between improving toxicities and potential anti-tumor effect of CAR-T cell.

Of note, in Axi-cel therapy, due to the previous tumor-related immunosuppression, lymphodepletion with fludarabine and cyclophosphamide, and the unique toxicity of CAR-T cell, subsequent immune reconstitution, B cell dysplasia and resultant hypogammaglobulinemia, patients are at high risk for infection complications (34–38). Although there were many other factors, such as CRS, ICANS, tocilizumab use, and bridging therapy may be associated with infection events, glucocorticoid, as an immunosuppressive agent, has been shown to increase the risk of severe and unusual infections (37, 39, 40). A recent study by Neill et al. demonstrated that glucocorticoids use was significantly associated with higher risk of infection, and rapid steroid taper was necessary (41). Infestations following CAR-T therapy which include bacterial, viral, fungal, and protozoal infection are dangerous and even fatal in these patients with weakened immune systems, so it is important to emphasize the importance of immunological monitoring to guide the strategies of antimicrobial prophylaxis for these patients, especially those with glucocorticoids management (34, 35, 39–43). In addition to infection, prolonged use of glucocorticoids may affect mental state, blood pressure, blood sugar and other vital physiological indicators, so for those who could not effectively respond to glucocorticoid in time, alternative strategies such as intrathecal cytotoxic chemotherapy and siltuximab that has a small molecular size than tocilizumab could be considered to avoid increasing glucocorticoid-related side effects (39, 44, 45).

We acknowledge as a major limitation of our review the non-randomized design in all included studies that led to a lack of adequate balance between cohorts, so there were many factors introducing bias and affecting the endpoints, the most important of which was the significant difference in the incidence and severity of CRS and ICANS between the two groups. Second, most of the studies we included are retrospective studies, and there is still a lack of eligible prospective studies. Third, although the impact of glucocorticoids management on CAR-T cell therapy is a concerning and urgent issue to be solved, due to the nature of CAR-T cell therapy and the lack of treatment methods for its associated toxicity, therefore, there are only one eligible clinical studies specifically explore the effects of glucocorticoids intervention on the efficacy of CAR-T cell therapy, and data on related factors that may impact the effects of glucocorticoids on the efficacy of CAR-T cell therapy are lacking, such as the timing, duration, and dose of glucocorticoids, age, tumor type, tumor burden, and complication of patient. Therefore, these findings should be interpreted with caution.

## Conclusion

Our study indicated that the association between the glucocorticoids use and the efficacy of CAR-T cell may be affected by cumulative dose, duration, and timing, among which timing is an urgent issue to be solved with conflicting results from different studies. Simple to glucocorticoids use as a variable may be detrimental to drawing a valid conclusion. Whether glucocorticoids should be recommended for early use and as a preventive treatment formulation in toxicity management needs to be cautious. It may be appropriate to comprehensively consider both the time to CAR-T cell infusion and toxicity grade. Moreover, because the glucocorticoids use can increase the risk of infection, it may be beneficial for patients to shorten the duration and wean doses rapidly if clinically feasible. In conclusion, a non-significant trend towards damaging the efficacy of CAR-T cell with glucocorticoids use should continue to be assessed in large prospective randomized controlled trials, and current evidence does not support the glucocorticoids use as a risk factor for impairing efficacy in Axi-cel therapy.

## Data Availability Statement

The original contributions presented in the study are included in the article/supplementary material. Further inquiries can be directed to the corresponding authors.

## Author Contributions

HQ, ZS and XW contributed to the conception and design of the study. RX, ZS searched the database. ZS and ML extracted data. ZS, RX, XW and ML wrote the manuscript. All authors contributed to the article and approved the submitted version.

## Conflict of Interest

The authors declare that the research was conducted in the absence of any commercial or financial relationships that could be construed as a potential conflict of interest.
